# Borderline Symptoms at Age 12 Signal Risk for Poor Outcomes During the Transition to Adulthood: Findings From a Genetically Sensitive Longitudinal Cohort Study

**DOI:** 10.1016/j.jaac.2019.07.005

**Published:** 2020-10

**Authors:** Jasmin Wertz, Avshalom Caspi, Antony Ambler, Louise Arseneault, Daniel W. Belsky, Andrea Danese, Helen L. Fisher, Timothy Matthews, Leah S. Richmond-Rakerd, Terrie E. Moffitt

**Affiliations:** a Duke University, Durham, North Carolina; b Duke University Medical Center, Durham, North Carolina; h Frank Porter Graham Child Development Institute, University of North Carolina, Chapel Hill; c Social, Genetic & Developmental Psychiatry Center, Institute of Psychiatry, Psychology and Neuroscience, King’s College London, United Kingdom; d Dunedin Multidisciplinary Health and Development Unit, University of Otago, New Zealand; e Columbia University Mailman School of Public Health, New York; f Institute of Psychiatry, Psychology and Neuroscience, King’s College London, United Kingdom; g National & Specialist CAMHS Clinic for Trauma, Anxiety and Depression, South London & Maudsley NHS Foundation Trust, London, United Kingdom

**Keywords:** adolescence, borderline, longitudinal, personality, twin

## Abstract

**Objective:**

Borderline personality disorder in adolescence remains a controversial construct. We addressed concerns about the prognostic significance of adolescent borderline pathology by testing whether borderline symptoms at age 12 years predict functioning during the transition to adulthood, at age 18 years, in areas critical to life-course development.

**Method:**

We studied members of the Environmental Risk (E-Risk) Longitudinal Twin Study, which tracks the development of a birth cohort of 2,232 British twin children. At age 12, study members' borderline symptoms were measured using mothers’ reports. At age 18, study members’ personality, psychopathology, functional outcomes, and experiences of victimization were measured using self-reports, coinformant reports, and official records.

**Results:**

At age 18, study members who had more borderline symptoms at age 12 were more likely to have difficult personalities, to struggle with poor mental health, to experience poor functional outcomes, and to have become victims of violence. Reports of poor outcomes were corroborated by coinformants and official records. Borderline symptoms in study members at 12 years old predicted poor outcomes over and above other behavioral and emotional problems during adolescence. Twin analyses showed that borderline symptoms in 12-year-olds were influenced by familial risk, particularly genetic risk, which accounted for associations with most poor outcomes at age 18.

**Conclusion:**

Borderline symptoms in 12-year-olds signal risk for pervasive poor functioning during the transition to adulthood. This association is driven by genetic influences, suggesting that borderline symptoms and poor outcomes are manifestations of shared genetic risk.

Borderline personality disorder is characterized by pervasive instability in a person’s mood, sense of self, impulse control, and interpersonal relationships. In adults, borderline personality disorder is considered a valid diagnosis by most clinicians.^1^ In adolescents, the diagnosis is more controversial.^2^, ^3^ Although diagnostic classification systems allow for a diagnosis of borderline personality disorder in adolescence, clinicians are reluctant to assess and treat borderline symptoms prior to adulthood.^4^, ^5^ Among the reasons cited for this reluctance are concerns that adolescents’ borderline symptoms may be transient; that a diagnosis could be stigmatizing; that personality development is still in flux; and that some borderline symptoms, such as impulsivity and difficulty in establishing a sense of identity, are inseparable from what is thought to be a normative degree of storm and stress during adolescence.^4^ In this study, we addressed concerns about the validity of adolescent borderline pathology by testing the prognostic significance of borderline symptoms in 12-year-old adolescents for psychosocial adjustment during the transition to adulthood, at age 18 years.

In recent years, research has made great strides toward establishing the validity of the borderline personality disorder diagnosis in adolescents. This research shows that borderline symptoms can be observed and reliably measured in adolescents, that symptoms are as prevalent in adolescents as they are in adults, that symptoms are relatively stable across time, and that symptoms predict a diagnosis of borderline personality disorder in adulthood.^2^, ^6^, ^7^, ^8^ Studies also report significant psychosocial impairment in adolescents who experience borderline symptoms.^9^ Another approach to testing the validity of borderline personality pathology is to examine the significance of adolescent borderline symptoms for adult adjustment. Previous findings suggest that adolescents who display borderline symptoms experience adjustment difficulties in adulthood.^10^ However, studies investigating the clinical and psychosocial outcomes of adolescent borderline symptoms are sparse, and a recent review of the literature concluded that many of these studies are limited by problems such as sampling bias, high attrition, and a narrow range of psychosocial outcomes.^11^ The aim of our study was to extend previous research by drawing a comprehensive picture of how adolescents with borderline symptoms fare during the transition to adulthood. Seven years ago, we described predictors and correlates of borderline symptoms measured in 12-year-old study members of the Environmental Risk (E-Risk) Longitudinal Twin Study, a population-representative birth cohort of twins born in the United Kingdom.^12^ Study members have now been followed up to age 18, with high retention (93%). At age 18, we assessed study members’ performance on a wide range of outcomes in areas critical to positive life-course development: personality functioning, mental health, functional outcomes, and experiences of victimization. Using these data, we tested the hypothesis that adolescent borderline symptoms predict poor outcomes during the transition to adulthood.

In addition to analyzing implications of borderline symptoms in 12-year-olds for outcomes at age 18, we tested whether symptoms contributed to poor outcomes independently of comorbid adolescent psychopathology and familial risk. We tested the role of comorbid psychopathology to investigate whether borderline symptoms demonstrate incremental validity beyond common disorders that clinicians assess in adolescents who present with emotional and behavioral dysregulation, such as conduct disorder, depression, and anxiety. Previous studies, including our own, show that many adolescents who display borderline symptoms also experience symptoms of these other disorders.^7^, ^12^ We tested the incremental validity of age 12 borderline symptoms by accounting for comorbid behavioral and emotional problems when evaluating effects of adolescent borderline symptoms on age 18 outcomes.

We tested the role of familial risk because adolescent borderline behaviors are strongly influenced by risk factors originating in families, both environmental and genetic.^12^, ^13^ Familial risk factors implicated in adolescent borderline symptoms, such as harsh parenting, maltreatment, and genetic susceptibility, also predict psychosocial adjustment in young adulthood.^13^, ^14^ These findings raise the possibility that poor outcomes are not due to adolescent borderline symptoms themselves, but that symptoms index familial risk for poor outcomes. We tested this hypothesis by comparing outcomes at age 18 within genetically identical twin pairs growing up in the same family who differed in adolescent borderline symptoms when assessed at age 12. Because these twins share all of their family-wide environment and genes, these analyses effectively control for familial risk factors shared between members of a family.

## Method

### Participants

Participants are members of the Environmental Risk (E-Risk) Longitudinal Twin Study, which tracks the development of a birth cohort of 2,232 British children.^15^ Briefly, the E-Risk sample was constructed in 1999−2000, when 1,116 families (93% of those eligible) with same-sex 5-year-old twins participated in home-visit assessments. This sample comprised 56% monozygotic (MZ) and 44% dizygotic (DZ) twin pairs; sex was evenly distributed within zygosity (49% male sex). The study sample represents the full range of socioeconomic conditions in Great Britain, as reflected in the families’ distribution on a neighborhood-level socioeconomic index (ACORN [A Classification of Residential Neighborhoods], developed by CACI, Inc., London, UK, for commercial use):^16^, ^17^ 25.6% of E-Risk families live in “wealthy achiever” neighborhoods compared with 25.3% nationwide; 5.3% compared with 11.6%, in “urban prosperity” neighborhoods; 29.6% compared with 26.9%, in “comfortably off” neighborhoods; 13.4% compared with 13.9%, in “moderate means” neighborhoods; and 26.1% compared with 20.7%, in “hard-pressed” neighborhoods. “Urban prosperity” neighborhoods are underrepresented in E-Risk because such households are often childless.

Home visits were conducted when the children were 7 years old (98% participation), 10 years old (96%), 12 years old (96%), and 18 years old (93%). At ages 5, 7, 10, and 12 years, assessments were carried out with participants as well as their mothers (or primary caretakers); the home visit at age 18 included interviews with participants only. Each twin was assessed by a different interviewer. These data are supplemented by searches of official records and by questionnaires that are mailed, as developmentally appropriate, to teachers and coinformants nominated by participants themselves. There were no differences between participants who did and did not take part at age 18 in terms of socioeconomic status assessed when the cohort was initially defined (χ^2^ = 0.86, *p* = .65), age 5 IQ scores (*t* = 0.98, *p* = .33), age 5 behavioral and emotional problems (*t* = 0.40, *p* = .69 and *t* = 0.41, *p* = .68, respectively) or age 12 borderline symptoms (*t* = 0.30, *p* = .76) The Joint South London and Maudsley and the Institute of Psychiatry Research Ethics Committee approved each phase of the study. Parents gave informed consent, and twins gave assent between age 5 and 12 years and then informed consent at age 18.

### Assessment of Borderline Symptoms

When study members were 12 years old, we collected information on their borderline symptoms during interviews with mothers, using items from the Shedler-Westen Assessment Procedure 200-item Q-Sort for Adolescents (SWAP-200-A)^5^ supplemented with items from the Achenbach System of Empirically Based Assessment.^18^ Items were selected from the set of SWAP-200-A items most commonly used by a sample of 294 doctoral-level clinicians to describe adolescent patients meeting *DSM-IV* diagnostic criteria for adult borderline personality disorder (Table 1).^19^ Of the 15 items selected from the SWAP, 5 items were very similar to items on the Achenbach scales used in E-Risk (eg, the SWAP item “Tends to be angry or hostile” was similar to the Achenbach scale item “Angry and hostile”). In these cases, we used the Achenbach scales item instead of the SWAP item to avoid asking mothers to rate the same item twice. All items and their descriptive statistics are reported in Table 1. Mothers were asked how well each item described their child (0, not true; 1, somewhat or sometimes true; 2, very true or often true). Data were available for 2,141 (99.8%) of participating members at age 12. Item responses were summarized into two measures that have been previously developed and described.^12^ First, a dimensional borderline symptoms scale was computed by summing up across items, with an internal consistency reliability of α = .86 (mean, 4.24; SD, 4.54; range, 0–26). We used this measure in our main analyses. Second, for illustrative purposes and to approximate clinically significant levels of borderline symptoms, we created a dichotomous measure identifying study members scoring at or above vs below the 95th percentile of the continuous borderline symptom scale at age 12 (*n* = 122, 5.7% of the sample). The 5% (or 2 SD) cutoff was chosen a priori because it is consistent with previous approaches to identifying clinically significant borderline pathology using a dimensional measure,^10^ falls within the range of prevalence estimates reported for clinically significant borderline pathology in adolescents,^20^ and is consistent with estimates of the prevalence of borderline personality disorder in adults in the community.^21^

**Table 1:** Mothers’ Ratings of Offspring’s Borderline Symptom Items in Adolescence, at Age 12 Years

Statement	Statement is very true or often true of child		
	Full sample	Boys	Girls
Easily jealous	9.0%	11.1%	6.9%
Falls for new friends intensely, expects too much too quickly	5.7%^a^	5.5%	5.6%
Changes friends constantly, loves them one day and hates the next	4.3%	4.7%	3.8%
Fears they will be rejected or abandoned	3.0%	3.2%	2.8%
Feels others are out to get him/her	2.1%	2.7%	1.5%
Acts overly seductive or sexy, flirts a lot	1.7%^a^	2.6%	0.9%
Attracted to unsuitable romantic partners	0.7%	0.7%	0.6%
Emotions spiral out of control, has extremes of rage, despair, excitement	9.1%^a^	10.5	7.7%
Cannot think when upset, becomes irrational	6.6%	7.5%	5.8%
Unable to soothe or comfort self	3.7%	4.0%	3.4%
Lacks stable image of self, changes goals/values	3.5%	3.8%	3.1%
Expresses emotions in an exaggerated dramatic way	11.5%	10.8%	12.2%
Irritable, touchy, or quick to “fly off the handle”	7.3%^a^	8.9%	5.7%
Angry and hostile	1.7%	2.2%	1.3%
Engages in self-harm behavior	2.9%	2.9%	2.9%

Note: N = 2,139–2,141.

a Sex differences were statistically significant at *p* < .05.

### Assessment of Outcomes at Age 18 Years

When study members were 18 years old, we collected information on a variety of outcomes indicating psychosocial adjustment: personality functioning, mental health, functional outcomes, and experiences of victimization (Table 2). We assessed outcomes using study members’ self-reports, reports by coinformants nominated by each twin (typically their cotwin and a parent), and official records. Outcomes and their assessment are described in Table 2.

**Table 2:** Description of Outcomes Measured During the Transition to Adulthood, at Age 18 Years

Measure	Informant	Description	Prevalence, %	Reference
**Personality**	Coinformants	At age 18, study members nominated two people “who knew them well.” Coinformants—mostly parents and cotwins—described each study member using a 25-item version of the Big Five Inventory. 99.3% of study members had data from at least one coinformant. 83% had data from 2 coinformants. We standardized and averaged data from coinformants.	—	40, 41
**Poor mental health**				
Mental disorder diagnoses				
Conduct disorder	Participant	Based on *DSM-5* criteria, assessed as part of a computer-assisted module.	15.1	42, 43
Alcohol use disorder	Participant	Based on *DSM-5* criteria, evaluated in face-to-face interviews using DIS.	27.8	42, 43
Cannabis use disorder	Participant	Based on *DSM-5* criteria, evaluated in face-to-face interviews using DIS.	6	42, 43
Depression	Participant	Based on *DSM-5* criteria, evaluated in face-to-face interviews using DIS.	20.1	42, 43
Generalized anxiety disorder	Participant	Based on *DSM-5* criteria, evaluated in face-to-face interviews using DIS. The 6-month symptom duration criterion was not required because of the young age of the study sample.	7.4	42, 43
Posttraumatic stress disorder	Participant	Based on *DSM-5* criteria, evaluated in face-to-face interviews using DIS.	4.4	42, 43
Suicide attempts or self-harm	Participant	To assess suicide attempts, study members were asked whether they had tried to kill themselves or attempted suicide since age 12, using a life-history calendar. If they answered positively, further questions were asked to obtain details and establish intent to die. 3.8% of study members had attempted suicide. To assess self-harm, study members were asked whether they had ever tried to hurt themselves, to cope with stress or emotional pain, since age 12, using a life-history calendar. Individuals who endorsed self-harm were queried about methods. 10 behaviors were probed (eg, cutting, burning, overdose), plus the option to describe any other way they had hurt themselves. 13.6% of study members had harmed themselves.	14.3	28, 44
Service use for behavioral or emotional problems	Participant	At age 18, study members reported whether they had accessed support services (eg, mental health professionals, medical doctors, or social services), spent time in the hospital, or had taken medication for dealing with emotional or behavioral problems in the past year.	17.2	^45^
Coinformant reports of poor mental health	Coinformants	Coinformants completed a questionnaire that included 10 items querying about study members’ mental health within the previous 12 months (example items: “Feels depressed, miserable, sad, or unhappy”; “Has alcohol problems”). We created a binary measure indicating whether both coinformants had endorsed one or more symptoms of poor mental health.	30.4	
**Poor functional outcomes**				
Low educational qualifications	Participant	Indicates whether study members reported that they did not obtain or scored a low average grade on their GCSE, a standardized examination taken at the end of compulsory education at age 16 years	21.9	
NEET status	Participant	Indicates whether study members were NEET, based on reporting that they were not studying, working in paid employment, or pursuing a vocational qualification or apprenticeship.	11.6	^46^
Cigarette smoking	Participant	Indicates whether study members reported that they were currently smoking daily.	22.3	
Risky sexual behavior	Participant	Indicates whether study members reported that they had engaged in two or more of the following risky sexual behaviors: having had sex before age 16; having had three or more sexual partners; practicing safe sex only sometimes or never; usually or always having sexual intercourse after a night out involving a lot of alcohol and/or drug use; having been told by a doctor that they had a sexually transmitted disease; and having had sexual relations resulting in pregnancy.	25.8	^47^
Social isolation	Participant	Study members were asked about their access to supportive relationships with family and friends using the MSPSS. The scale scoring was reversed, and social isolation was defined as being among the 20% highest scoring study members.	20.0	48, 49
Low life satisfaction	Participant	Study members were asked about their life satisfaction using the Satisfaction With Life Scale. The scale scoring was reversed, and low life satisfaction was defined as being among the 20% highest scoring study members.	18.7	49, 50
Official crime record	Official records	Official records of participants’ cautions and convictions beginning at age 10 through age 19 were obtained through United Kingdom Police National Computer record searches conducted in cooperation with the Ministry of Justice.	10.2	^49^
**Victimization during adolescence**	Participant	Participants were interviewed about exposure to a range of adverse experiences between 12 and 18 years using the JVQ-R2, adapted as a clinical interview. Exposure to victimization was coded on a 3-point scale (0, no exposure; 1, probable or less severe exposure; 2, definite or severe exposure). Individuals who reported a definite or severe level of exposure were coded as positive. Our adapted JVQ comprised 45 questions covering 7 different forms of victimization: maltreatment (3.3%), neglect (2.2%), sexual victimization (2.6%), family violence (12.1%), peer/sibling victimization (15.6%), cyber-victimization (6.5%), and crime victimization (19.3%).		51, 52, 53

Note: DIS = Diagnostic Interview Schedule; GCSE = General Certificate of Secondary Education; JVQ-R2 = Juvenile Victimization Questionnaire, 2nd revision; MSPSS = Multidimensional Scale of Perceived Social Support; NEET = not in education, employment, or training; PNC = Police National Computer.

### Covariates: Adolescent Behavioral and Emotional Problems and Childhood Victimization

Symptoms of conduct disorder at age 12 were measured using mothers’ and teachers’ reports of children’s behavioral problems, using the Achenbach family of instruments and *DSM-IV* items as previously described.^22^, ^23^, ^24^ Consistent with *DSM-IV* criteria, children with five or more symptoms were assigned a diagnosis of conduct disorder (5.5% of cohort). Depression and anxiety at age 12 were assessed using children’s self-reports on the Children’s Depression Inventory^25^ and the 10-item version of the Multidimensional Anxiety Scale for Children,^26^ respectively. Scores of 20 or more on the Children’s Depression Inventory were used to indicate clinically significant depressive symptoms^25^, ^27^ (3.5% of cohort), and scores of 13 or more (corresponding to the 95th percentile) on the Multidimensional Anxiety Scale for Children were used to indicate extreme anxiety^28^ (6.1% of cohort).

### Statistical Analyses

Our statistical analysis proceeded as follows. First, we tested associations between the continuous measure of borderline symptoms at age 12, standardized to mean (M) = 0, standard deviation (SD) = 1, and poor outcomes at age 18. We did this by predicting each poor outcome from age 12 borderline symptoms. All models included sex of study members as a covariate. We illustrate the results of these analyses by comparing percentages and means of poor outcomes at age 18 years among study members with a high vs lower adolescent borderline symptom score, defined as being at or above vs below the 95th percentile for borderline symptoms at age 12. Second, we tested whether borderline symptoms added incremental value to other behavioral and emotional problems that study members experienced at age 12. We did this by adding symptom scores of conduct disorder, depression, and anxiety at age 12 as additional covariates to test unique effects of adolescent borderline symptoms on poor outcomes. Third, we tested whether borderline symptoms were influenced by familial risk. We did this by comparing correlations in borderline symptoms among genetically identical (MZ; *n* = 594) and nonidentical (DZ; *n* = 476) twin pairs. We also formally analyzed genetic and environmental influences on adolescent borderline symptoms using a univariate twin model.^29^ Twin models compare within-pair similarity for MZ twins, who are genetically identical, and DZ twins, who share on average half their segregating genes. This information can be used to estimate genetic (A), shared environmental (C), and nonshared environmental (E) influences on a phenotype. C represents environmental factors that make members of a family similar, whereas E represents factors that make members of a family different and also includes error of measurement. Fourth, we compared poor outcomes among genetically identical twins who differed in their borderline symptoms at age 12 to test whether adolescent borderline symptoms predict poor outcomes over and above familial influences—both genetic and environmental—shared between identical twins growing up in the same family. Differences in borderline symptoms were operationalized as any difference in the continuous symptoms score between identical (MZ) twins. There were 462 MZ pairs who differed in their borderline symptom score at age 12.

Poisson regression models were used for binary outcomes, and linear regression models were used for continuous outcomes. We chose Poisson over logistic regression models for the binary outcomes to obtain risk ratios, which are a more easily interpretable measure of risk, particularly when outcomes are common. Standard errors were adjusted for the clustering of twins within families. Fixed-effects Poisson and linear regression models with robust standard errors were used for the twin comparisons. Stata version 14.1^30^ was used for these analyses. Twin models were fitted using the structural equation modeling program OpenMx.^31^

## Results

### Borderline Symptoms in 12-Year-Olds Predicted a Difficult Personality at Age 18

**Figure 1:**
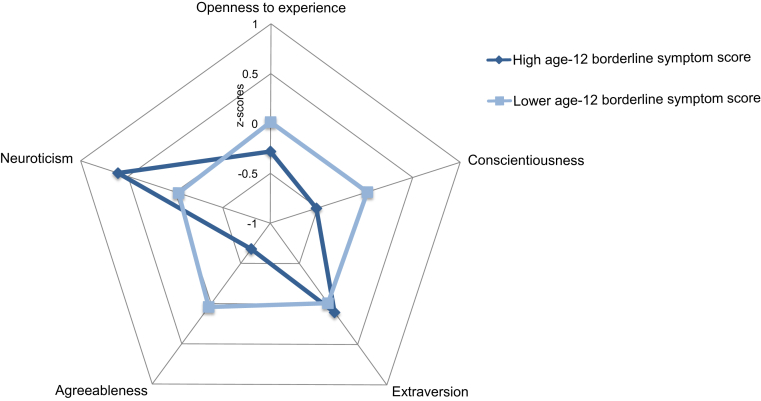
Personality Profiles for Individuals With High Borderline Symptom Scores vs Their Cohort Peers With Lower Symptom Scores ***Note:****A high borderline symptom score at age 12 (operationalized as being at or above the 95th percentile for borderline symptoms at age 12) predicts a distinct personality profile at age 18, characterized by narrow-mindedness (low openness to experience), antagonism (low agreeableness), distress (high neuroticism), and poor impulse control (low conscientiousness). All analyses are adjusted for study members’ sex. Please note color figures are available online.*

Study members with more borderline symptoms at age 12 showed a profile of more difficult personality at age 18 compared to their peers with fewer borderline symptoms. Specifically, coinformants who knew these study members well described them as more narrow-minded (low openness to experience), antagonistic (low agreeableness), easily distressed (high neuroticism), and as having poorer impulse control (low conscientiousness) at age 18 (Table 3). To approximate clinically significant levels of borderline symptoms, we created a categorical measure by grouping study members with high borderline symptom scores (operationalized as scoring at or above the 95th percentile of the quantitative symptom scale at age 12; *n* = 122, 5.7% of the sample). Figure 1 shows personality profiles for individuals with high borderline symptom scores vs their cohort peers with lower symptom scores.

**Table 3:** Borderline Symptoms (Measured on a Continuous Scale) of 12-Year-Olds Predict Poor Outcomes at Age 18

Age 18 outcome	Model 1^a^	Model 2^b^	Model 3^c^
**Personality**	β **(95% CI)**	β **(95% CI)**	β **(95% CI)**
Openness to experience	**−.08 (−0.13, −0.03)**	−.02 (−0.08, 0.04)	.00 (−0.10, 0.11)
Conscientiousness	**−.16 (−0.21, −0.12)**	**−.09 (−.14, −.03)**	.02 (−.11, .14)
Extraversion	**.06 (0.02, 0.11)**	**.13 (0.07, 0.18)**	.02 (−0.11, 0.14)
Agreeableness	**−.28 (−0.32, −0.23)**	**−.17 (−0.22, −0.11)**	−.08 (−0.17, 0.03)
Neuroticism	**.23 (0.19, 0.28)**	**.19 (0.13, 0.25)**	m.09 (−0.03, 0.22)
**Poor mental health**	**IRR (95% CI)**	**IRR (95% CI)**	**IRR (95% CI)**
Conduct disorder	**1.41 (1.31, 1.51)**	**1.17 (1.05, 1.31)**	1.02 (0.81, 1.29)
Alcohol use disorder	**1.12 (1.05, 1.19)**	1.08 (0.99, 1.17)	0.99 (0.81, 1.22)
Cannabis use disorder	**1.44 (1.24, 1.66)**	1.09 (0.89, 1.32)	1.04 (0.73, 1.49)
Depression	**1.18 (1.09, 1.28)**	1.09 (0.99, 1.20)	0.95 (0.79, 1.15)
Generalized anxiety disorder	1.13 (0.97, 1.31)	1.02 (0.83, 1.23)	1.32 (0.90, 1.92)
Posttraumatic stress disorder	**1.26 (1.05, 1.52)**	1.15 (0.90, 1.47)	1.61 (0.86, 2.99)
Suicide attempts or self-harm	**1.38 (1.27, 1.50)**	**1.26 (1.13, 1.40)**	1.10 (0.89, 1.35)
Service use	**1.31 (1.21, 1.41)**	**1.26 (1.14, 1.40)**	1.18 (0.99, 1.40)
Coinformant report of poor mental health	**1.36 (1.29, 1.44)**	**1.21 (1.12, 1.31)**	1.00 (0.87, 1.14)
**Poor functioning**	**IRR (95% CI)**	**IRR (95% CI)**	**IRR (95% CI)**
Low educational qualifications	**1.40 (1.32, 1.49)**	**1.18 (1.08, 1.28)**	1.13 (0.93, 1.38)
NEET status	**1.35 (1.21, 1.50)**	0.99 (0.86, 1.13)	1.12 (0.74, 1.68)
Cigarette smoking	**1.40 (1.33, 1.49)**	**1.19 (1.09, 1.30)**	1.00 (0.89, 1.11)
Risky sexual behavior	**1.29 (1.22, 1.37)**	**1.17 (1.07, 1.26)**	0.93 (0.78, 1.11)
Social isolation	**1.23 (1.14, 1.33)**	1.07 (0.97, 1.18)	1.14 (0.95, 1.37)
Low life satisfaction	**1.27 (1.17, 1.36)**	1.06 (0.96, 1.17)	1.14 (0.95, 1.37)
Official crime record	**1.54 (1.40, 1.69)**	**1.19 (1.03, 1.36)**	1.02 (0.84, 1.26)
**Adolescent victimization**	**IRR (95% CI)**	**IRR (95% CI)**	**IRR (95% CI)**
Maltreatment	**1.89 (1.61, 2.22)**	**1.47 (1.09, 1.98)**	1.18 (0.95, 1.46)
Neglect	**1.84 (1.56, 2.17)**	**1.63 (1.23, 2.16)**	1.10 (0.61, 1.98)
Sexual victimization	**1.45 (1.22, 1.72)**	1.00 (0.77, 1.29)	1.30 (0.80, 2.10)
Family violence	**1.30 (1.18, 1.44)**	1.14 (1.00, 1.31)	0.85 (0.66, 1.11)
Peer victimization	**1.28 (1.17, 1.39)**	**1.17 (1.05, 1.31)**	1.07 (0.87, 1.31)
Cyber-victimization	**1.32 (1.16, 1.50)**	**1.27 (1.07, 1.51)**	1.15 (0.73, 1.80)
Crime victimization	**1.25 (1.16, 1.34)**	**1.16 (1.05, 1.29)**	1.01 (0.84, 1.22)

Note: Boldface type indicates statistically significant estimates (*p* > .05). All outcome measures are described in Table 2. β = standardized regression coefficient; IRR = incidence rate ratio (interpretable as risk ratios); NEET = not in education, employment, or training.

a Model 1: Analyses adjust for sex. For models additionally adjusting for baseline measures where possible, see Table S1, available online.

b Model 2: Analyses additionally adjust for age 12 behavioral and emotional problems.

c Model 3: Estimates are within-monozygotic-twin-pair estimates, ie, analyses adjust for the influence of genes and environments shared between genetically identical twins growing up in the same family.

### Borderline Symptoms in 12-Year-Olds Predicted Poor Mental Health at Age 18

**Figure 2:**
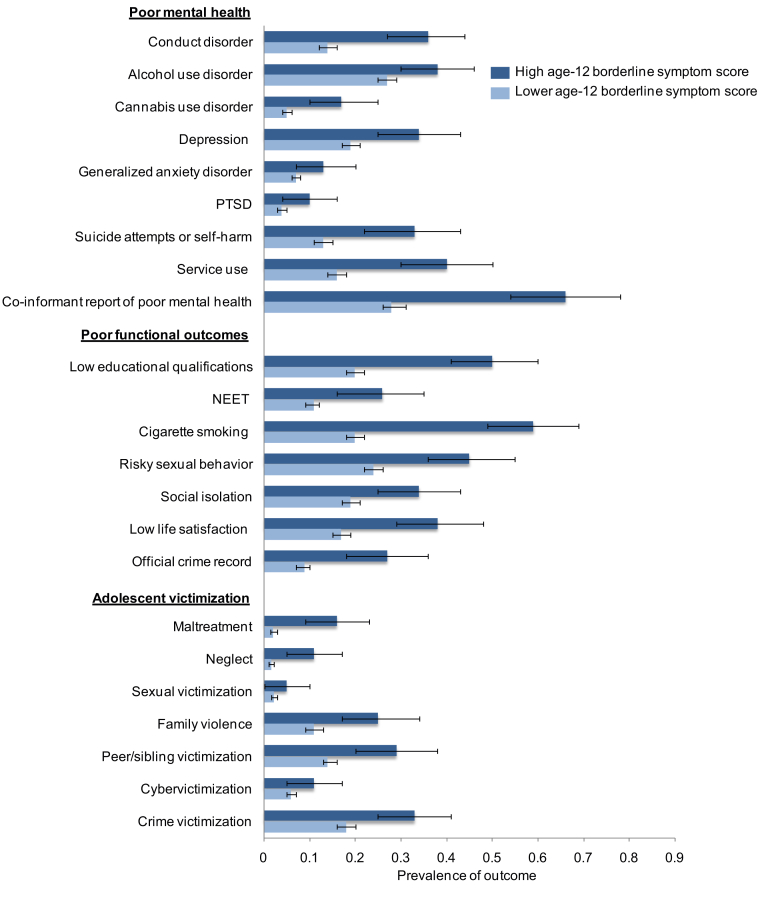
Prevalence of Mental Health Outcomes Among Study Members With High vs Lower Borderline Symptom Scores at Age 12 ***Note:****12-year-olds with a high borderline symptom score (operationalized as being at or above the 95th percentile for borderline symptoms at age 12) experience worse outcomes at age 18 compared with their cohort peers with a lower borderline symptom score. Error bars indicate 95% CIs. All analyses adjust for study members’ sex. All outcome measures are described in*Table 2*. NEET = not in education, employment, or training; PTSD = posttraumatic stress disorder. Please note color figures are available online.*

Study members with more borderline symptoms at age 12 experienced worse mental health at age 18 compared to their peers with fewer symptoms (Table 3). They were more likely to meet diagnostic criteria for a mental disorder, to have attempted suicide or engaged in self-harm, and to have used clinical and support services to cope with emotional and behavioral problems. Findings of worse mental health were corroborated by coinformants (Table 3). These findings are illustrated in Figure 2, which shows the prevalence of mental health outcomes among study members with high vs lower borderline symptom scores at age 12.

### Borderline Symptoms in 12-Year-Olds Predicted Poor Functioning at Age 18

Study members with more borderline symptoms at age 12 experienced worse functioning at age 18 compared to their peers with fewer symptoms (Table 3). They had poorer educational and economic outcomes, as indicated by educational failure and unemployment; engaged in more health-risk behaviors, as indicated by cigarette smoking and risky sexual activity; experienced lower well-being, as indicated by social isolation and dissatisfaction with life; and were more likely to have broken the law, as indicated by having an official crime record (Table 3). These findings are illustrated in Figure 2, which shows the prevalence of poor functional outcomes among study members with high vs lower borderline symptom scores at age 12.

### Borderline Symptoms in 12-Year-Olds Predicted Becoming a Victim of Violence

Study members with more borderline symptoms at age 12 were more likely to become victims of violence during adolescence (age 12–18 years) compared to their peers with fewer symptoms (Table 3). Adolescents with borderline symptoms experienced victimization both within and outside of their families through maltreatment, neglect, family violence, bullying by peers, and as victims of crime (Table 3). These findings are illustrated in Figure 2, which shows the prevalence of victimization exposures among study members with high vs lower borderline symptom scores at age 12. Previous studies, including our own, have shown that adolescent borderline symptoms are often preceded by victimization during childhood.^12^ Victimization during adolescence may therefore reflect continuing exposure to victimization rather than effects of borderline symptoms. However, even after statistically controlling for childhood victimization, borderline symptoms predicted adolescents’ risk of becoming victimized in adolescence (Table S1, available online).

### Borderline Symptoms in 12-Year-Olds Were Correlated With Behavioral and Emotional Problems, But This Did Not Explain Away Associations With Most Poor Outcomes

At age 12, study members who displayed more borderline symptoms tended to also experience more symptoms of other behavioral and emotional problems, including conduct disorder (*r* = .56, 95% CI [0.51, 0.62], *p* < .01), depression (*r* = .27, 95% CI [0.21, 0.32], *p* < .01), and anxiety (*r* = .10, 95% CI [0.04, 0.15], *p* < .01). More than half (55%) of study members with a high (ie, at or above the 95th percentile) borderline symptom score at age 12 met clinical criteria for at least one of these problems compared with 10% of study members with a lower symptom score (ie, below the 95th percentile). We tested whether borderline symptoms added incremental value to behavioral and emotional problems when predicting poor outcomes by statistically controlling for continuous symptom scores of conduct disorder, depression, and anxiety at age 12 when predicting poor outcomes (Table 3). Borderline symptoms continued to predict most outcomes independently of correlated problems, particularly a difficult personality at age 18, and most of the poor functional outcomes and experiences of victimization. Some associations between age 12 borderline symptoms and age 18 poor outcomes were explained away by co-occurring behavioral and emotional problems at age 12, most notably associations with nearly all diagnoses of mental disorders at age 18. However, even within the psychiatric outcomes domain, borderline symptoms in 12-year-olds continued to predict adverse outcomes, including conduct disorder, suicide attempts and self-harm, service use, and coinformant reports of poor mental health (Table 3).

### Borderline Symptoms in 12-Year-Olds Developed Against a Backdrop of Familial Risk, Which Accounted for Most Associations With Most Poor Outcomes at Age 18

Twins growing up in the same families resembled each other in their borderline symptom scores at age 12 (*r* = .49, 95% CI [0.42, 0.55], *p* < .01), suggesting familial risk for borderline symptoms. Comparing correlations between members of genetically identical (MZ) and nonidentical (DZ) twin pairs revealed that familial risk was entirely genetic, as indicated by MZ correlations that were twice as high as DZ correlations (*r*_*MZ*_ = .66, 95% CI [0.60, 0.73], *p* < .01; *r*_*DZ*_ = .28, 95% CI [0.19, 0.37], *p* < .01). We formally analyzed twin correlations using a univariate twin model and obtained a heritability estimate (A) of 0.66 (95% CI [0.58, 0.70]), indicating that 66% of individual differences in borderline symptoms at age 12 were explained by genetic influences (Table S2, available online). The remainder was accounted for by environmental influences not shared between family members (E) (estimate: E = 0.34; 95% CI [0.30, 0.38]). There were no shared environmental influences (C) (estimate: C = 0.00; 95% CI [0.00, 0.07]). If genetic influences affect both borderline symptoms at age 12 and poor outcomes at age 18, adolescent borderline symptoms may be an expression of shared genetic risk for poor outcomes, rather than an influential factor in itself. Our findings supported this hypothesis: genetically identical twins who differed in borderline symptoms experienced similar levels of poor outcomes at age 18 (Table 3). This finding suggests that borderline symptoms in 12-year-olds predict poor outcomes at age 18 because borderline symptoms and poor outcomes are manifestations of shared genetic risk.

## Discussion

Our follow-up of 12-year-olds with borderline symptoms to age 18 revealed three main findings. First, at a time in life when young people take a leap toward greater social, economic, and personal maturity, study members with a history of borderline symptoms were held back by psychosocial difficulties. Difficulties were evident in numerous areas (personality; psychopathology; vocational, health, and social functioning; and experiences of victimization); observed by multiple informants; and assessed through multiple methods, including official records. Differences in outcomes were striking: young people with the highest borderline symptom scores at age 12 were nearly three times more likely to engage in suicidal and self-harming behavior; to find themselves without training or job opportunities; to have a criminal record; and to have experienced victimization compared to their cohort peers with lower symptom scores. These findings show that adolescent borderline symptoms observed as early as at age 12 forecast meaningful individual differences in young people’s lives.

Second, although many 12-year-olds experienced behavioral and emotional problems alongside their borderline symptoms, borderline symptoms added incremental value to predicting most poor outcomes over and above these other problems, indicating that the later-life impairments associated with adolescent borderline pathology are insufficiently described by these problems. Notably, behavioral and emotional problems of 12-year-olds accounted for associations with nearly all of their psychiatric diagnoses at age 18, but did not account for associations with many other adverse outcomes. This finding illustrates that psychiatric diagnoses do not capture the full scope of life challenges associated with adolescent borderline symptoms and shows that it is important to look beyond psychiatric status when testing the predictive validity of adolescent borderline symptoms.

Third, borderline symptoms of 12-year-olds were under considerable genetic influence, and genetically identical twins of children with elevated borderline symptoms were at increased risk for poor outcomes even if they did not have equally elevated borderline symptoms themselves. This finding raises three issues. First, it raises the question of why twins with the same genetic susceptibility do not share similar borderline symptoms. Our previous study pointed to twins’ unique environmental experiences as a possible explanation: we reported that twins in the same families experienced different levels of harsh parental treatment and that adolescents’ genetic vulnerability interacted with harsh parental treatment in the etiology of borderline symptoms.^12^ This finding is consistent with diathesis-stress models of borderline personality, which propose that it is the interaction between children’s genetically influenced, early emerging temperamental difficulties and an invalidating, abusive, and ineffective caregiving environment that increases risk for borderline problems (and other poor outcomes) in a transactional process across development.^12^, ^32^ Second, our findings indicate that adolescent borderline symptoms reflect broader genetic risk for poor outcomes, rather than being the cause of these outcomes. This finding does not undermine the prognostic significance of adolescent borderline symptoms. Rather, it suggests that adolescents remain at risk for adverse psychosocial outcomes even after symptom reduction.^10^ Third, if borderline symptoms are not the cause of poor outcomes but are on the pathway from genetic risk to poor outcomes, more work is needed to understand how genetic risk influences both borderline symptoms and poor outcomes. A hypothesis consistent with diathesis-stress models of borderline personality is that genetic risk begins to manifest early in life, as a difficult temperamental profile characterized by high negative affect, poor impulse control, and high emotional sensitivity. A child’s difficult temperament subsequently increases risk for borderline pathology as well as for other poor outcomes, particularly when it is met by an invalidating caregiving environment.^32^

Our work expands on previous literature in three ways. First, although several studies have investigated the clinical and psychosocial outcomes of borderline personality disorder, a recent systematic review concluded that many of these studies have limitations, such as sampling bias, high rates of attrition, and a narrow range of psychosocial outcomes.^11^ Our study overcomes some of these limitations because our cohort is nationally representative, follow-up of participants has occurred with extremely low attrition, and we report associations with a wide range of clinical and psychosocial outcomes. Second, there are very few studies testing associations between borderline pathology and exposure to victimization, particularly in adolescence. Our study extends the literature by showing that borderline symptoms in 12-year-olds predict exposure to different types of victimization, both inside and outside the home, during adolescence. Third, in addition to reporting that adolescents’ borderline pathology predicts poor outcomes, we find that these associations do not persist after accounting for familial influences shared between identical twins growing up in the same family. Although several studies have tested outcomes of adolescent borderline symptoms using a twin design,^33^ to our knowledge our study is the first to apply this approach to a wide range of clinical and psychosocial outcomes.

Our findings should be interpreted in light of some limitations. First, we did not make a formal diagnosis of borderline personality disorder. Without a replication in adolescents with a diagnosis of borderline personality disorder, we cannot be sure that our findings generalize to this population. However, our measure captures core diagnostic features of borderline personality disorder (affective instability, cognitive disturbance, impulsivity, and interpersonal dysfunction), and our previous study showed that the etiological factors, comorbidity, sex differences, and heritability of our measure of borderline symptoms are comparable to results from studies of borderline personality disorder in community samples.^10^, ^12^, ^34^, ^35^ Second, a general weakness of discordant twin analyses is their higher likelihood of false-negative findings because the limited variation within twin pairs magnifies the impact of measurement error and reduces the precision of estimates.^36^ Third, our study does not contain a measure of borderline symptoms at age 18, so we were unable to test the continuity of borderline symptoms. However, our findings show that 18-year-olds with a history of borderline symptoms show a personality profile characterized by emotional and interpersonal lability that is typical of adults with borderline personality disorder.^37^ Fourth, although a considerable portion of 12-year-olds with borderline symptoms went on to experience poor outcomes, there were also adolescents who bucked this trend and fared well despite their symptom history. Follow-on work is needed to investigate factors that predict variability in poor outcomes associated with borderline symptoms. Fifth, our study members are still young, so it is unclear how persistent their psychosocial difficulties will be. However, many of the outcomes we measured—low educational qualifications, cigarette smoking, personality dysfunction, having a criminal record, risky sexual behavior—are still meaningful because they represent barriers to leading a prosperous and healthy adult life. Moreover, other studies that have tested associations with some of the same outcomes we report, such as attainment and social support, has shown that adolescent borderline symptoms predict these outcomes up to age 33 years.^10^

Our findings have implications for health professionals working with adolescents who display borderline symptoms. First, our findings support the assessment of adolescents’ borderline symptoms in addition to other emotional and behavioral disorders if borderline pathology is suspected. Some clinicians are thought to prefer assessing only emotional and behavioral disorders in adolescents presenting with borderline symptoms, perhaps to avoid a stigmatizing diagnosis of personality disorder.^6^, ^38^ However, our findings show that adolescents’ borderline symptoms provide independent prognostic information. Second, our findings argue in favor of early access to treatment for adolescents with borderline symptoms and against a wait-and-see approach.^39^ Psychological treatments for adult patients with borderline personality disorder have been adapted for use with adolescents and show promise for improving symptoms.^7^ In addition to treatment for personality pathology, adolescents with borderline symptoms need access to support services that help reduce the risk for future poor functioning, such as educational support services. Third, our findings show that adolescents’ borderline symptoms signal a longer-term need for care. Even if symptoms decrease after treatment, adolescents remain at risk for adverse outcomes because symptoms partly reflect genetic risk for future difficulties. Adolescents should be monitored and supported accordingly, particularly during the transition to adulthood when they face discharge from child and adolescent mental health services. Fourth, our findings imply that young people with a history of borderline symptoms may turn up on the doorsteps of many services, including mental health care services, unemployment offices, sexual health centers, courts, emergency departments, and social services. The breadth of poor outcomes among these young people requires an integrated treatment approach that involves coordination across multiple social and support services.

## Figures and Tables

**Table S1:** Borderline Symptoms (Measured on a Continuous Scale) at Age 12 Predict Poor Outcomes at Age 18 Even After Adjusting for Poor Adjustment During Childhood

Age 18 outcome	Model 1^a^	Model 2^b^
**Personality**	β **(95% CI)**	β **(95% CI)**
Openness to experience	**−.08 (−0.13, −0.03)**	**−.07 (−0.12, −0.02)**
Conscientiousness	**−.16 (−0.21, −0.12)**	**−.14 (−0.19, −0.09)**
Extraversion	**.06 (0.02, 0.11)**	**.07 (0.02, 0.12)**
Agreeableness	**−.28 (−0.32, −0.23)**	**−.24 (−0.29, −0.20)**
Neuroticism	**.23 (0.19, 0.28)**	**.23 (0.19, 0.28)**
**Poor mental health**	**IRR (95% CI)**	**IRR (95% CI)**
Conduct disorder	**1.41 (1.31, 1.51)**	**1.30 (1.18, 1.42)**
Alcohol use disorder	**1.12 (1.05, 1.19)**	N/A
Cannabis use disorder	**1.44 (1.24, 1.66)**	N/A
Depression	**1.18 (1.09, 1.28)**	**1.14 (1.05, 1.24)**
Generalized anxiety disorder	1.13 (0.97, 1.31)	1.12 (0.97, 1.30)
Posttraumatic stress disorder	**1.26 (1.05, 1.52)**	N/A
Suicide attempts or self-harm	**1.38 (1.27, 1.50)**	**1.31 (1.19, 1.43)**
Service use	**1.31 (1.21, 1.41)**	**1.27 (1.18, 1.38)**
Coinformant report of poor mental health	**1.36 (1.29, 1.44)**	N/A
**Poor functioning**	**IRR (95% CI)**	**IRR (95% CI)**
Low educational qualifications	**1.40 (1.32, 1.49)**	**1.23 (1.15, 1.31)**
NEET status	**1.35 (1.21, 1.50)**	N/A
Cigarette smoking	**1.40 (1.33, 1.49)**	N/A
Risky sexual behavior	**1.29 (1.22, 1.37)**	N/A
Social isolation	**1.23 (1.14, 1.33)**	1.07 (0.97, 1.18)
Low life satisfaction	**1.27 (1.17, 1.36)**	N/A
Official crime record	**1.54 (1.40, 1.69)**	N/A
**Adolescent victimization**	**IRR (95% CI)**	**IRR (95% CI)**
Maltreatment	**1.89 (1.61, 2.22)**	**1.66 (1.39, 1.98)**
Neglect	**1.84 (1.56, 2.17)**	**1.59 (1.34, 1.88)**
Sexual victimization	**1.45 (1.22, 1.72)**	**1.26 (1.07, 1.50)**
Family violence	**1.30 (1.18, 1.44)**	**1.20 (1.09, 1.33)**
Peer victimization	**1.28 (1.17, 1.39)**	**1.25 (1.14, 1.36)**
Cyber-victimization	**1.32 (1.16, 1.50)**	**1.29 (1.12, 1.48)**
Crime victimization	**1.25 (1.16, 1.34)**	**1.19 (1.10, 1.28)**

Note: Boldface type indicates statistically significant estimates (*p* > .05). β = standardized regression coefficient; IRR=incidence rate ratio (interpretable as risk ratios); N/A = not applicable.

a Model 1: Models are adjusted for sex only.

b Model 2: Models are additionally adjusted for the baseline measurement of the respective outcome (eg, when predicting personality functioning at age 18, we adjusted for personality ratings by examiners at age 12; when predicting attainment at age 18, we adjusted for educational attainment as rated by teachers at age 12; when predicting victimization during adolescence, we adjusted for childhood physical or sexual victimization by an adult as assessed through mothers’ reports, as previously described.^1^, ^2^ Adjusting for the baseline measurement was not always possible (eg, because outcomes could not or were not measured during childhood, such as risky sexual behavior).

**Table S2:** Genetic and Environmental Influences on Borderline Symptoms at Age 12

	Variance components		
	A (95% CI)	C (95% CI)	E (95% CI)
Age 12 borderline symptoms	0.66 (0.58, 0.70)	0.00 (0.00, 0.07)	0.34 (0.30, 0.38)

Note: A = additive genetic influences; C = shared environmental influences; E = nonshared environmental influences .
